# Optimizing Performance in Badminton Tournaments: The Relationship Between Timing, Quantity, and Quality Among Professional Players

**DOI:** 10.3390/jfmk10010005

**Published:** 2024-12-27

**Authors:** Jen-Hao Hsu, Hsin-Lun Lin, Hung-Chieh Fan Chiang, Duan-Shin Lee, Yang Lee, Cheng-Wei Huang, Zai-Fu Yao

**Affiliations:** 1 Physical Education Office, National Tsing Hua University, Hsinchu City 30013, Taiwan; jenhao@mx.nthu.edu.tw; 2 Department of Interdisciplinary Program of Science, National Tsing Hua University, Hsinchu City 30013, Taiwan; hsinlun0409@gmail.com; 3 Department of Physics, National Tsing Hua University, Hsinchu City 30013, Taiwan; 4 Institute of Communications Engineering, National Tsing Hua University, Hsinchu City 30013, Taiwan; 5 Department of Sports Training Science-Balls, National Taiwan Sport University, Taoyuan City 33325, Taiwan; 6 Department of Athletics, National Taiwan University of Science and Technology, Taipei City 106335, Taiwan; 7 College of Education, National Tsing Hua University, Nanda Campus, No. 521, Nanda Rd., Hsinchu City 300193, Taiwan; 8 Research Center for Education and Mind Sciences, National Tsing Hua University, Hsinchu City 30013, Taiwan; 9 Department of Kinesiology, National Tsing Hua University, Hsinchu City 30013, Taiwan; 10 Basic Psychology Group, Department of Educational Psychology and Counseling, National Tsing Hua University, Hsinchu City 30013, Taiwan

**Keywords:** tournament scheduling, performance optimization, badminton, Olympic qualification strategy, sports simulation modeling

## Abstract

**Background/Objectives:** Top badminton players must carefully schedule tournaments to perform well and improve their rankings. This study examines how players plan their tournament participation and whether their scheduling strategies affect their rankings and performance, especially during Olympic and non-Olympic years. **Methods:** Data were extracted from the Badminton World Federation (BWF) ranking system for the top 50 men’s and women’s singles players from May 2014 to May 2019. A computer-based simulation model and cluster analysis were applied to analyze tournament participation patterns, recovery intervals, and performance trends. Statistical analyses were conducted to identify correlations between these variables and ranking outcomes. **Results:** Top-ranked players participated in more tournaments during Olympic years, with those competing in more than nine tournaments achieving better results. Players performing well in higher-graded tournaments tended to take longer recovery breaks, whereas lower-performing players entered more tournaments. Cluster analysis revealed two distinct performance groups, with the top 20 men’s and top 12 women’s singles players adopting different strategies compared to lower-ranked counterparts. **Conclusions:** Strategic scheduling of tournaments, including balancing participation frequency and recovery intervals, is vital for optimizing performance and achieving sustained ranking success. These findings emphasize the importance of high-graded tournament selection and tailored schedules for elite players.

## 1. Introduction

Badminton is considered one of the most fiercely competitive sports worldwide [1,2], with rankings varying across the World, BWF World Tournament, and Olympic qualification. However, during Olympic years, most players earned more points by participating in tournaments than in non-Olympic years, since the quartile range in 2016 was 15 to 24, while in other years, it was about 12 to 16. This observation means that players who participate in more tournaments during Olympic years have a better chance of earning more points and climbing higher in the world rankings. The primary means of assessing top-level players’ quality is through the badminton world rankings, which are determined by the cumulative points earned over the preceding 52 weeks. In line with this notion, this study analyzes rankings based on the data provided by the Badminton World Federation (BWF) as of May 5th annually, from 2014 to 2019. This approach aligns with the Olympic ranking criteria during Olympic years, ensuring an accurate and relevant analysis for our research. However, badminton players may adopt different strategies to earn more points and achieve a higher ranking by avoiding playing against seeded players in early matches during Olympic years. This strategy is commonly referred to as “strategic tanking”. This strategy can be especially effective during the Olympics [3] when players earn more points by participating in more tournaments. By avoiding higher-ranked opponents in early rounds, players can enter more tournaments and have a better chance of earning more points overall, which can improve their ranking. Yet, this strategic arrangement can backfire if a player loses too many early matches, and their ranking drops too low. The consequence of such an arrangement can make it more challenging to enter high-level tournaments in the future, limiting their opportunities to earn more points and improving their ranking. Also, excessive tournament participation without adequate recovery can lead to over-fatigue or overtraining, potentially reducing performance and increasing the risk of injury [4,5,6,7,8]. It should be noted that the number of tournaments played does not necessarily correspond with a higher ranking. Instead, players who advance in competitions and participate in better-graded tournaments earn more points. Therefore, it is still being determined whether fluctuations in these rankings truly reflect players’ performances. Nevertheless, excessive tournament participation can result in fatigue, burnout, or injury, undermining efficiency and performance [2,9,10].

Recent studies [11,12,13,14] have emphasized the importance of recovery and strategic tournament selection in elite sports performance. Elite athletes often adopt block periodization and ATR (accumulation, transformation, realization) model approaches in their preparation, which focus on alternating cycles of high-intensity training and recovery. For example, block periodization and ATR models are commonly employed by elite athletes to alternate high-intensity training and recovery phases, enabling them to maintain peak performance during critical competitions [15]. These strategies allow athletes to manage fatigue effectively while peaking during critical competitions. By combining these methods, athletes can strategically include lower-level tournaments for skill refinement and recovery periods between high-stakes events, ensuring optimal performance at key moments. Therefore, Athletes employing these strategies may alternate between competitions of varying importance while ensuring adequate recovery, thereby optimizing both short-term and long-term performance outcomes. Such preparation could be a critical factor differentiating high-performing players from their lower-ranked counterparts.

A tournament’s score is based on its level, significance, and the player’s performance [4]. The badminton ranking system utilizes a player’s top 10 highest-scoring events within the preceding year to determine their ranking. If a player participates in more than ten events during this period, such as 12, the two lowest-scoring events are excluded from the calculation. However, only the first nine highest-scoring tournaments a player participates in within the past 52 weeks should be considered as “countable tournaments”. These events carry the most weight since they are the most recent and relevant in assessing a player’s state of play, skill level, and career trajectory. As players compete in additional tournaments, the significance of the counting events gradually diminishes. Consequently, this study examines the correlation between the number of tournaments played by players, their ranking, and performance variations to ascertain whether the best performance in nine tournaments over the previous year can predict their ranking.

### Research Questions and Aims

As far as we know, only one prior study, for judo [16], has looked into the best strategy for selecting the ideal time interval between tournaments for top players [1]. However, this research area remains under-explored in badminton. Therefore, this study investigates whether there is an ideal interval between tournaments for athletes to recover from their last competition and whether a strategy for selecting the ideal time interval between tournaments to play among the 50 top-ranked men’s and women’s singles players impacts their performance ranking. This study diverges from conventional approaches by employing computational simulation and a greedy algorithm to model tournament scheduling and ranking strategies in professional badminton. In line with this, this study also investigates whether a player’s best-performed nine tournaments over the past 52 weeks could have predictive values of their ranking and whether the number of tournaments that athletes played in can account for their ranking and explain performance variances. Specifically, we test whether top-ranked singles players exhibited different strategies or patterns of ideal and actual game performance than lower-ranked singles players. Lastly, the results explore whether the strategic planning of tournament schedules differs for top-ranked and low-ranked singles players and whether the number of tournaments played during the qualification period affects the singles players’ ranking and their chances of qualifying for major tournaments like the Olympics [1,3,17]. The present study aims to define what constitutes an elite or top-ranked badminton singles player, mainly focusing on the criteria utilized to distinguish between top and low-ranked singles players. Specifically, this study aims to ascertain the cutoff points that separate elite players from their low-ranked counterparts while providing the quantitative matrices that underpin these criteria. The matrices under consideration include points earned, rankings, and others. Ultimately, the findings aim to provide a deeper understanding of the contextual factors, such as recovery time or tournament selection, that influence the classification of elite badminton players.

## 2. Materials and Methods

### 2.1. Study Design

This study employed a computational-analytical and simulation-based design to examine the relationship between tournament scheduling, rest intervals, and player rankings in badminton. Specifically, this study combined retrospective data analysis with simulation modeling to assess real-world player performance during the Olympic qualification period (2014–2018) and a simulation year (2019).

### 2.2. Data Collection and Description

In this computational-analytical and simulation-based design study, since the computational simulation and sampling approaches are based on BWF ranking system, we extracted data from the BWF official page (https://bwf.tournamentsoftware.com/ranking/ranking.aspx?rid=70) for every top player who submitted to the BWF’s ranking system from May 2014 to May 2019. The data only included those men’s singles and women’s singles players who were ranked in the top 50 and played in Grade 1 events, such as the Olympics and the World Championships, or Grade 2 tournaments, such as the Super Series (1000, 750, 500, 300, 100) and the World Tour Finals, during Olympic and non-Olympic years. Specifically, 2016 was the Olympic years that we discussed. Thirty-eight tournaments were documented for analysis, including ten tournaments in the Super 100, twelve in the Super 300, six in the Super 500, five in the Super 750, and four in the Super 1000. Players earned ranking points by participating in and winning matches in tournaments [18], with higher levels tournaments offering higher ranking points. Only players ranked up to 150 qualified to participate in the Grade 2 competition, and the top 15 players were obligated to attend all Super 1000 and 750 tournaments and at least four Super 500 tournaments. Generally, higher-ranked players receive more points for tournament performance than lower-ranked players. The reason for choosing this criterion is because the BWF rankings, which determine Badminton Olympic qualification, are based on the number of points earned within a specific qualification window. Grade 2 events, including Level 1—BWF World Tour Finals and Level 2—Asian Championships, or above (i.e., Grade 1 events such as the BWF World Championships/Olympics), offer ranking points to players based on their performance in different rounds. Super Series tournaments also provide ranking points, with Super Series Premier tournaments offering more points, second only to BWF tournaments. The points earned determine the top eight players who qualify for the Super Series Finals and World Rankings. However, the number of tournaments players participate in can affect their ranking and points. It is possible for higher-ranked badminton players to join fewer tournaments but still earn similar points or reach the same level of performance as lower-ranked players. This postulation is because the BWF ranking system is based on a player’s performance in tournaments over 52 weeks, with more recent performances carrying more significant weight. During the 52-week Olympic qualification period, players must amass enough ranking points to be eligible for the Olympic Games. The top-ranked players have the potential to earn more points than in other years due to the increased competition and significance of the Olympic Games. Consequently, players must carefully plan their tournament schedules to maximize their chances of earning enough points to qualify. Top-ranked players may focus on major tournaments, such as the Olympics, while lower-ranked players may participate in more tournaments to secure enough points to qualify. While injuries may significantly impact a player’s ability to participate in tournaments, this study did not account for injuries as a variable due to the limitations in publicly available data on athlete health status. We acknowledge that injuries, such as those experienced by players like Carolina Marin (e.g., ACL injuries), can influence the frequency of tournament participation and, consequently, their performance and ranking. Although the data did not allow for injury-related controls, future research may benefit from incorporating this factor to better assess its potential confounding effects on player performance metrics. This study used the highest ranking each year as an outcome measure for quantifying player performance over winning percentage, given that the points earned for each grade of the tournament differed, and a player with a similar winning percentage could have a significantly varied career-high ranking.

### 2.3. Variables and Sampling

In this study, our analysis primarily focused on sampling athletes from the top 50 rankings to determine various parameters. Initially, athletes between rankings 2–8, followed by one of every fifth athlete after that, were sampled to assess the intervals between competitions.

#### 2.3.1. Variables Studied

Dependent Variables: Interval of weeks between competitions.

Independent Variables: Athletes’ rankings and participation throughout the past 52 weeks.

#### 2.3.2. Sampling

The data collection process involved systematic sampling from the top 50 rankings of the 2019 men’s and women’s singles.

Additionally, more tournaments were considered based on which tournament earned minor points in the nine effective tournaments and used the number of games played in the tournament at a level one round lower than the minimum points counted in that tournament as a model for the points earned in different levels of the Badminton World Federation’s grade system; such a tournament was considered to be an energy-wasted tournament. Lastly, resampling by a hybrid of simple and systematic sampling was conducted based on the data in the first part.

The tournament parameters, specifically the number of games played, were used as a model to determine the points earned at different levels within the Badminton World Federation’s grade system.

### 2.4. General Descriptive Statistical Analysis

The statistical analysis in this study involved a combination of descriptive statistics, simulation modeling, and comparison of real and simulated data to assess the relationship between tournament scheduling, rest intervals, and player performance rankings.

Descriptive Statistics:

Initial analyses included calculating the mean and standard deviation of rest intervals between tournaments for the top 50 men’s and women’s singles players. This provided a baseline understanding of how frequently players participated in tournaments and allowed for a comparison between top-ranked and lower-ranked players.

Simulation and Algorithm:

A program was developed using the greedy algorithm, implemented through C++ compiled by GCC, to simulate an ideal point-ranking distribution with two constraints: the current highest-ranked player obtains the current highest point, and each player will participate in only nine tournaments.

Comparison of Simulated and Actual Data:

The analysis also involved analyzing the difference between the results of the simulated ideal situation and the actual data to understand the relationship between real and ideal scenarios.

Linear Regression Models:

In this study, we applied a linear regression model to examine the relationship between the number of tournaments played and player rankings. The model’s fit was assessed using R^2^ (R-squared or the coefficient of determination), a statistical measure in regression analysis that represents the proportion of variance in the dependent variable that can be explained by the independent variable. In other words, R^2^ indicates how well the data fit the regression model, with higher values reflecting stronger goodness of fit and a greater proportion of ranking variability being explained by tournament participation. Additionally, we used the variance inflation factor (VIF) [19], a statistical measure that qualifies how much the variance of regression coefficients is inflated due to collinearity among predictors, to ensure that the regression model’s coefficients were not inflated due to correlations among the variable. Homogeneity of variance was evaluated using Levene’s test [20], which assesses the equality of variances across groups to ensure that the residuals were evenly distributed across all levels of the independent variable. To assess the adequacy of the regression model, a residual analysis was performed. Residuals, defined as the differences between observed and predicted values, provide insights into the model’s performance and the validity of the assumptions underlying the regression analysis. The analysis involved plotting residuals against the predicted values to check for patterns that might indicate non-linearity, heteroscedasticity, or other violations of regression assumptions. A random scatter of residuals around zero would suggest that the model is appropriate for the data. Additionally, a normal Q-Q residual plot was examined to assess their normality, as regression analysis assumes that residuals are normally distributed. These tests confirmed that the regression assumptions were adequately met for reliable analysis.

Software and Tools:

The statistical analyses were carried out using R (version 4.4.2) for descriptive statistics, regression analysis, and ANOVA, while C++ was employed for simulation modeling. For additional statistical testing, SPSS (IBM Corp. Released 2023. IBM SPSS Statistics for Windows, Version 29.0.2.0 Armonk, NY, USA: IBM Corp) and Prism (GraphPad Prism version 9.0.0 for Windows) were used. All statistical tests assumed a significance level of *p* < 0.05.

## 3. Results

### 3.1. What Is the Ideal Time Interval Between Tournaments for Top Athletes to Recover from Their Previous Competitions to Limit Their Participation in Subsequent Tournaments?

Based on the data, it was found that the average rest time between tournaments for top men’s singles players who quenched was 5.04 weeks, whereas those who annealed had an average rest time of 5.06 weeks. It can also be observed that the average rest time between tournaments for the top women’s singles players who quenched was 4.45 weeks. However, the standard deviation of time intervals between tournaments with top-ranked players ranged from 2–8 weeks, suggesting that the top-ranked players have varied tournament schedules compared to lower-ranked players. Moreover, the standard deviation for quenched players was 4.33 weeks for top men’s singles and 3.47 weeks for top women’s players.

**Figure 1:**
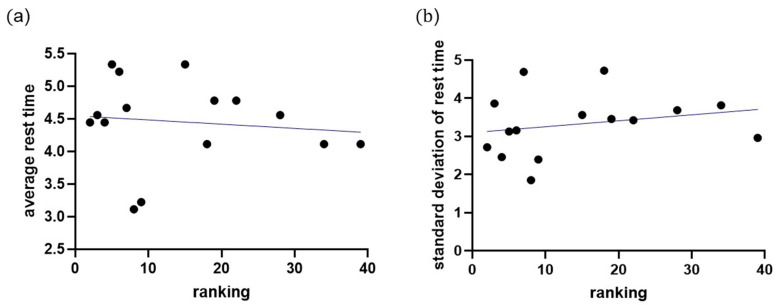
(**a**) The relation between average rest time (week) and men’s singles players’ ranking. (**b**) The relation between the standard deviation of rest time (week) and men’s singles players’ ranking. The dot indicates the average time interval between tournaments, and the bar indicates the standard deviation of the data. In addition to ideal points, we compute the ideal time interval (rest time) between tournaments, as shown in Figure 1.

**Figure 2:**
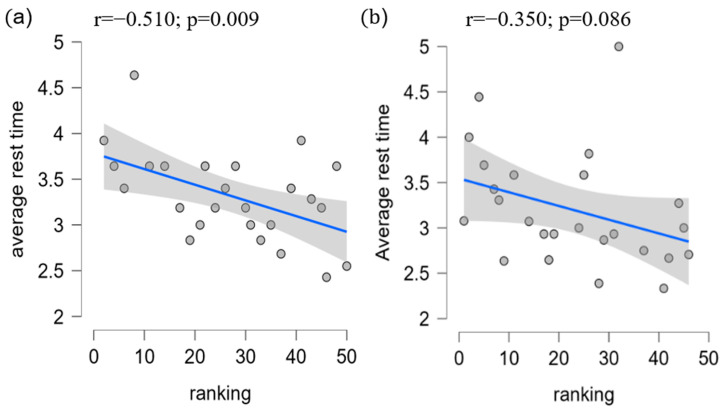
Negative correlation between ranking and new average rest time based on 2019 in men’s/women’s singles with 95% confidence interval. (**a**) = men’s singles; (**b**) = women’s singles. *p* = *p*-value; r = Pearson’s r.

**Figure 3:**
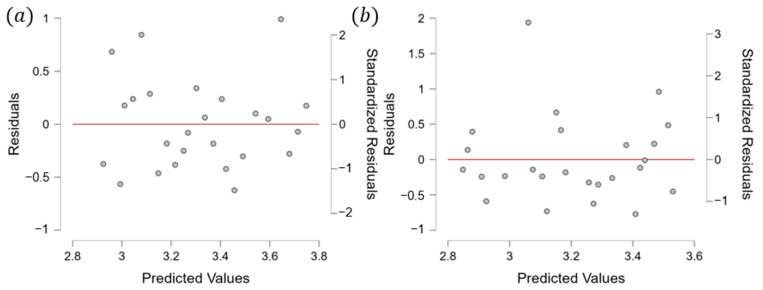
A residual plot for men’s and women’s singles. (**a**) Residual plot for men’s singles. (**b**) Residual plot for women’s singles.

**Figure 4:**
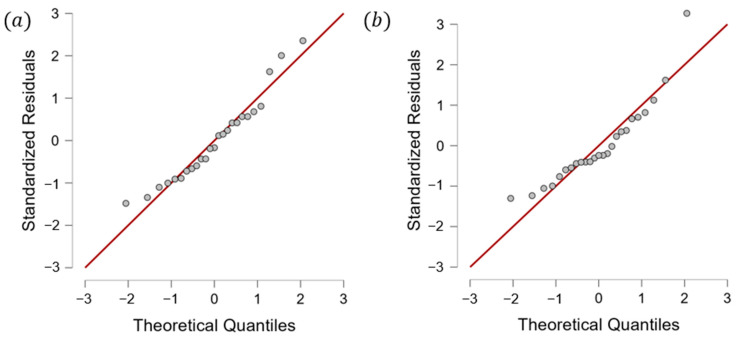
Q-Q plots for men’s and women’s singles. (**a**) Normal Q-Q plot for men’s singles. (**b**) Normal Q-Q plot for women’s singles.’

**Figure 5:**
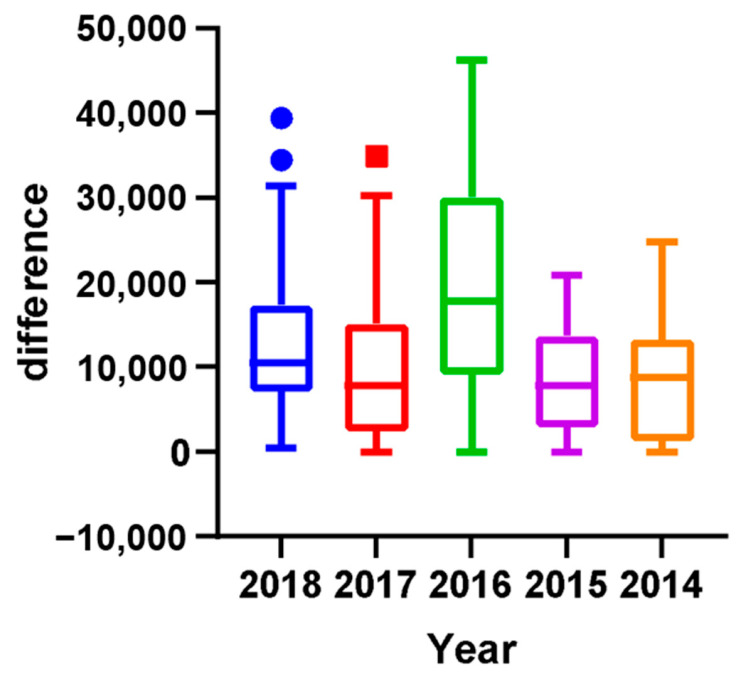
Total points earned in tournaments by the top 50 men’s players in Olympic and non-Olympic years. Note: the circle and the squares indicate outliers (out of the 1.5 interquartile range).

**Figure 6:**
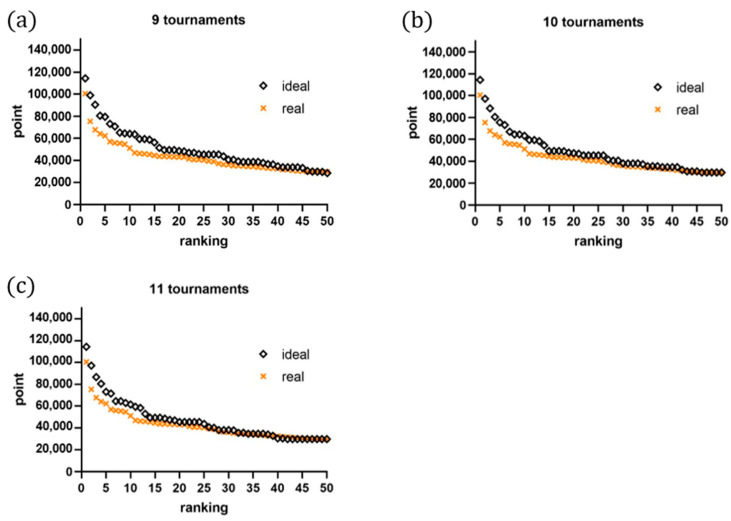

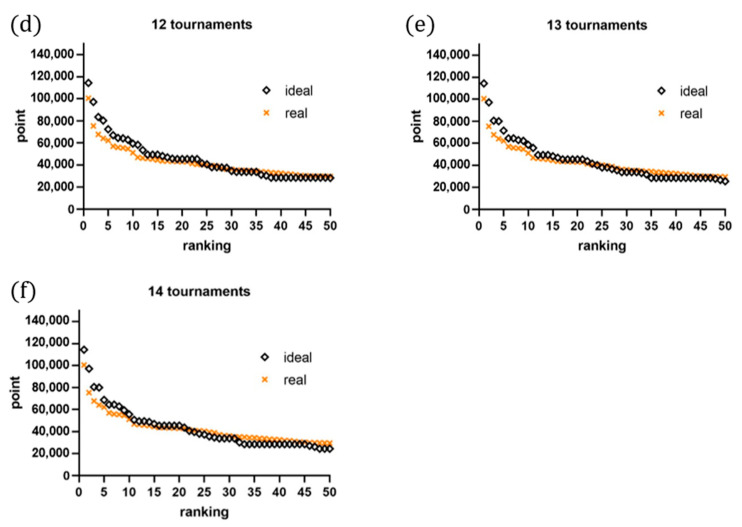
(**a**) The simulation of ideal points earned corresponding to the ideal ranking in nine tournaments played against actual points earned and ranking performances. (**b**–**f**) same as (**a**) but 10, 11, 12, 13, and 14 tournaments, respectively.

**Figure 7:**
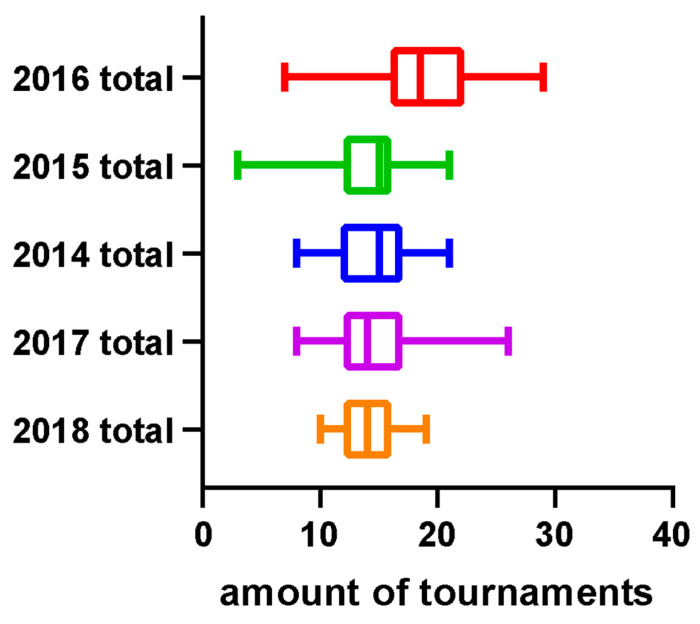
The differences between the total number of tournaments for men’s players across Olympic and non-Olympic years.

Specifically, the annealed players had the same rest weeks as the quenched players. These findings indicate that single-top players tend to space out their tournaments for adequate recovery time and to avoid excessive fatigue, burnout, or injury. Additionally, players who performed well in higher-graded tournaments tended to take longer breaks between tournaments to recover fully. By contrast, those who performed less well in these tournaments tended to participate in more tournaments to accumulate more points. Although this study did not identify the ideal recovery time, it highlighted that athletes could not attend every competition and must depend on strategies to space out their tournaments (see Figure 1). Moreover, to analyze the relationships between tournament participation, recovery intervals, and player performance, we applied a linear regression model. The regression analysis revealed a significant negative correlation between ranking and average rest intervals for men’s singles players (r = −0.31, *p* < 0.05), with an R^2^ value of 0.26, indicating that 26% of the variance in rankings could be explained by rest intervals. Women’s singles players demonstrated a similar trend (r = −0.21, *p* < 0.05), with an R^2^ value of 0.11. The VIF values (range: 1.12–1.35) confirmed no multicollinearity, and Levene’s test (*p* = 0.08) validated the homogeneity of variances. Figure 2 presents these findings, illustrating the inverse relationship between rest intervals and player rankings. This model allowed us to assess the correlation between the number of tournaments played and player rankings, with *p*-values < 0.05 considered statistically significant. The results indicated a negative correlation between player ranking and rest intervals, demonstrating that lower-ranked players tend to have shorter recovery periods between tournaments (Figure 2). Figure 2 illustrates the regression analysis results, showing a significant inverse relationship between rest intervals and player rankings, with R^2^ = 0.26 for men’s singles and R^2^ = 0.11 for women’s singles. Figure 2 presents the results of a linear regression analysis, showing a significant negative correlation between player ranking and the average time interval between tournaments. Figure 1 illustrates the observed average rest intervals and their variability, while Figure 2 presents the linear regression results, showing the correlation between rest intervals and rankings. Lower-ranked players tended to have shorter rest intervals, while higher-ranked players took longer breaks, especially when recovering from high-stakes tournaments. Moreover, the R squares of the model for men’s and women’s singles were 0.2601 and 0.1125, respectively, which led to the VIF (variation inflation factor) values between 1.3513 and 1.126. After that, we conducted Levene’s test to check homogeneity with *p*-value < 0.05. The VIF values ranged from 1.12 to 1.35, confirming no multicollinearity among predictors, while Levene’s test (*p* = 0.08) indicated homogeneity of variances. It showed that collinearity and homogeneity assumptions were also checked, with no violations found. Additionally, the low R^2^ values observed in the models for men’s singles and women’s singles indicate that the independent variables included in the regression model suggest that other influential factors not accounted for in this model, such as psychological variables, training regimens, or external conditions, may play a significant role in determining players’ performance and ranking outcomes. Therefore, a residual analysis was performed to evaluate the adequacy of the regression model. Residuals were plotted (Figure 3) against predicted values to ensure no discernible patterns, indicating that the model’s assumptions were valid. A Q-Q plot (Figure 4) of residuals confirmed their approximate normality, supporting the validity of the regression outcomes. This finding supports the observation that players who perform well in higher-graded tournaments often take extended recovery periods to optimize their performance in subsequent tournaments. Additionally, players who performed well in higher-graded tournaments tended to take longer breaks between tournaments to recover fully. The negative correlation in Figure 5 reinforces this conclusion, as lower-ranked players participate more frequently with shorter rest intervals. Detailed breakdowns of statistical outcomes, including descriptive statistics and regression coefficients, are provided in Figure 2, Figure 3, Figure 4, Figure 5, Figure 6 and Figure 7. However, to participate in more tournament does not directly lead to more fatigue. Therefore, further research, incorporating physiological and psychological measures of fatigue, would be necessary to evaluate the direct relationship between frequent participation and athlete well-being. This gap in the current analysis points to the need for exploring preparation and recovery strategies among different ranking groups better to understand their impact on performance and fatigue management.

Our study is delimited to analyzing the top 50 men’s and women’s singles players, which stems from two primary considerations:

(1) Data Accessibility: Retrieving comprehensive data for all players posed considerable challenges. Focusing on the top 50 allowed us to ensure a more robust and accurate analysis within the available data scope.
(2) Tournament Points Distribution: The distribution of earned points, notably pertaining to Grade 1 events, like the Olympics and the World Championships, or Grade 2 tournaments, such as the Super Series, significantly differs among players ranking 50 and below. Consequently, focusing on the top 50 provides a more concentrated and relevant dataset for our study objectives.

While this focus may limit our findings’ generalizability to the broader player population, it was instrumental in conducting a thorough and detailed analysis within the confines of available and reliable data sources.

### 3.2. What Is the Ideal Trade-Off Between the Number of Tournaments Played and Points Earned/Ranking Variances Explained for Increasing Efficiency? How It Influences the Point Distribution? Are There Differences in Tactic Strategies Between High-Ranked and Low-Ranked Players?

This study simulated a greedy algorithm with a single constraint to test the hypothesis that models the ideal relationship between points earned and expected rank in nine tournaments. The greedy algorithm assumes that all players aim to maximize their scores, with higher-ranked players having a greater likelihood of winning matches against lower-ranked opponents. Under this assumption, the algorithm enables each player to strategically select the best possible option to achieve their goals while adhering to the defined constraints. In the program, players are assigned scores sequentially until each player accumulates nine scores corresponding to the tournaments they participated in, ensuring that the scoring process is equitable and systematic. The simulated ideal points earned and ideal rankings were compared to the actual distribution of points earned by players who participated in more than nine tournaments. However, a discrepancy was observed between the predicted and actual data, resulting in some players’ exclusion of specific data points and a lower point allocation. Specifically, the results indicate that, while the model perfectly fit top-ranked players’ career progression, it was less accurate for lower-ranked players, revealing two distinct data clusters with a boundary around the 20th rank. A similar pattern was also found in women’s singles players, suggesting two distinct data clusters with a boundary around the 12th rank. Next, all athletes are categorized into two clusters: the top 20 and the remaining athletes. The model showed that the latter class fits perfectly with the ideal situation, while the former did not. Therefore, based on this analysis, the high-ranked players were defined as those who are within the top 20 with more lenient criteria according to our program. A simulation was plotted to illustrate the ideal points earned relative to the ideal ranking across different numbers of tournaments. The findings suggest that increasing the number of tournaments leads to more ideal results. Lastly, a figure was presented showing the prediction and simulation residuals for players at different numbers of tournaments played for all players (see Figure 6).

### 3.3. How Does the Strategic Allocation of Points by Top-Ranked Players in Tournaments Held in the Years Preceding Olympic Qualification Affect the Points Accrued by Lower-Ranked Players?

**Figure 8:**
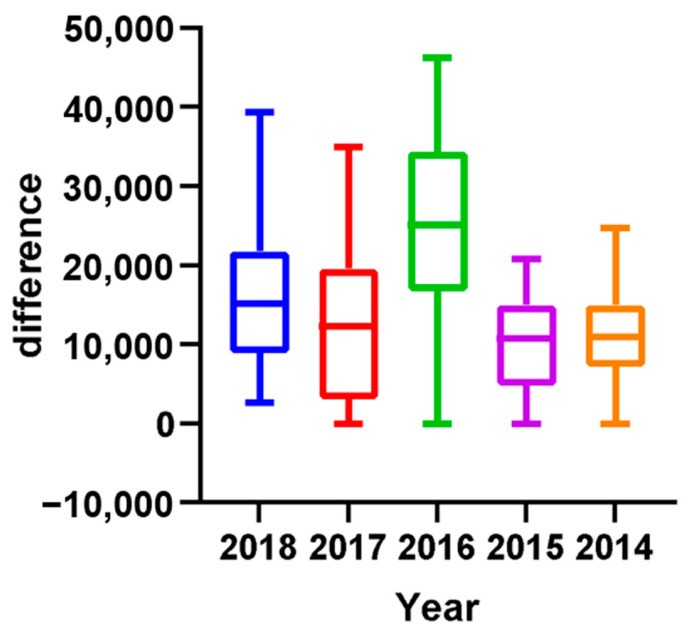
Difference between total points and actual points earned by the Top 32 men’s players in Olympic and non-Olympic years during the tournament’s participation.

**Figure 9:**
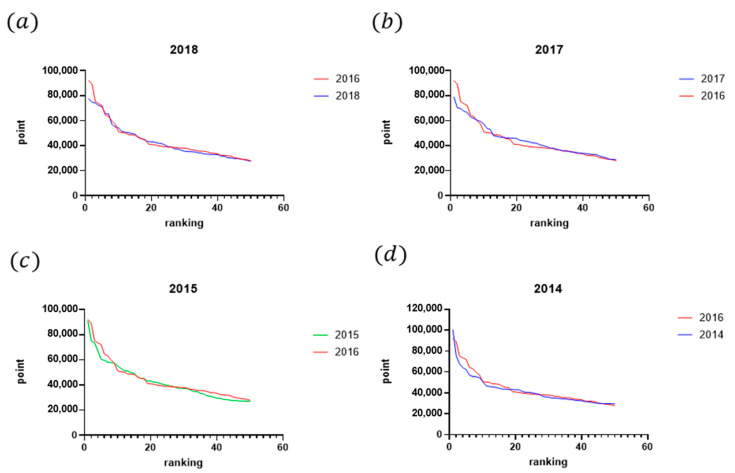
(**a**–**d**) The comparison between the Olympic year (2016) and non-Olympic years (2018, 2017, 2015, 2014), respectively.

**Figure 10:**
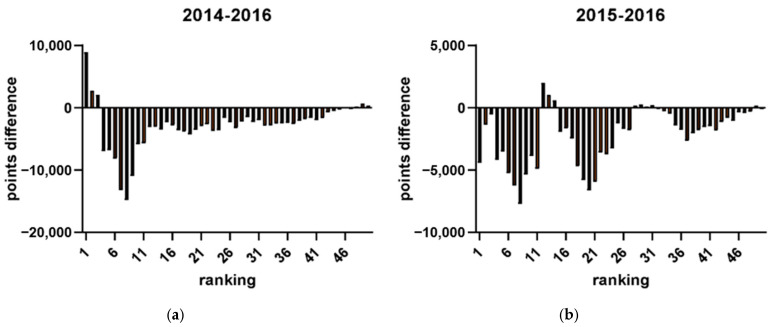

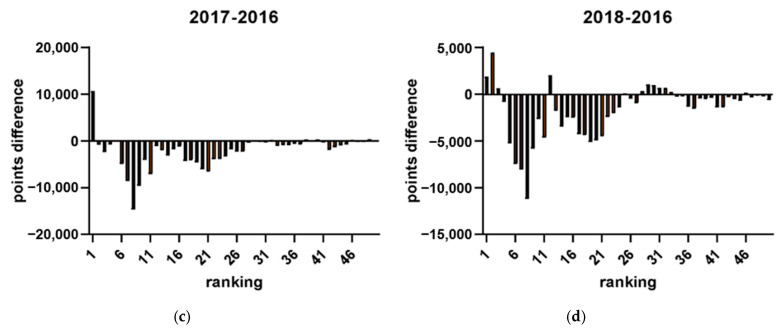
(**a**–**d**) The distribution of points earned and rankings in the top 50 men’s singles players across Olympic (2016) and non-Olympic (2014, 2015, 2017, 2018) years, respectively.

**Figure 11:**
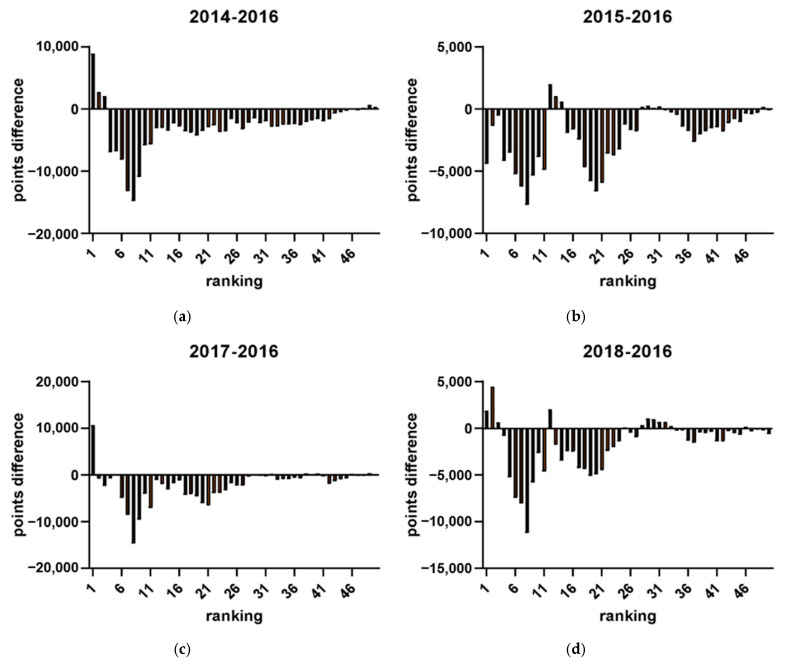
(**a**–**d**) The distribution of points earned and rankings in the top 50 women’s singles players across Olympic (2016) and non-Olympic years (2014, 2015, 2017, 2018), respectively.

A retrospective analysis of participation records from previous Olympic years was conducted to determine the distribution of players in the tournament bracket. The level of players within each ranking range was quantitatively assessed to identify their positions among the tournament participants. Calculations were made to demonstrate how the decline in points is affected by higher-ranked players (i.e., as defined by our findings from the last section: top 20 for men’s players and top 12 for women’s players). To investigate the impact of the Olympic strategy on the number of tournaments (see Figure 7), a comparison was made between the number of tournaments in Olympic and non-Olympic years. Additionally, the average number of tournaments among the top 32 players between Olympic and non-Olympic years was compared. The selection of 32 players as the criterion was based on the format of Super 1000 tournaments, which have no qualifying rounds, and include only 32 in their singles and doubles draws [21]. That is, these tournaments, such as the All England Open, the Indonesia Open, and the China Open are exclusive to the most elite players. To determine the effect of the Olympic strategy on points earned, a comparison was made between total points earned by all players and actual points earned by the top 32 players in Olympic and non-Olympic years during the participation of the tournament by plotting the figures (see Figure 8 and the example in Figure 9). Further analysis investigated the range of athletes affected by the Olympic strategy by plotting a distribution of points earned and rankings in the top 50 men’s singles players across Olympic and non-Olympic years (see Figure 10). A similar distribution was also plotted for the top 50 women’s singles players across Olympic and non-Olympic years (see Figure 11).

This study aimed to investigate the impact of the strategy employed by top-ranked players in accumulating points during tournaments leading up to Olympic qualification on the overall points earned by lower-ranked players. The analysis revealed that the number of tournaments was higher in Olympic years compared to non-Olympic years (see Figure 5). Furthermore, the top-ranked players participated in the highest average number of tournaments during Olympic years compared to non-Olympic years. The top 32 players also earned more total points during the Olympics than in non-Olympic years. However, the analysis showed that athletes ranking about 20 have fewer points in Olympic years compared to non-Olympic years. These findings are consistent with previous results, indicating that the model was highly effective in predicting the career progression of the top-ranked men’s players. Yet, it was less reliable for lower-ranked players. Two distinct data clusters were observed with a clear boundary at around the 20th rank. This pattern was also evident in women’s singles players, where two distinct clusters were identified with a boundary at approximately the 12th rank. Therefore, the cutoff rankings among the elite top-ranked 20 players within the ranking can be considered top-ranked. By contrast, the rest of the other ranked players can only be determined as top players. The findings suggest that the strategy employed by top-ranked players affects the overall points earned by lower-ranked players and can impact their rankings in the tournament bracket.

## 4. Discussion

This study examines world rankings based on the accumulation of points over the preceding 52 weeks, focusing on Grade 2 events or above. Grade 2 events offer ranking points to men’s and women’s singles badminton players based on their performance in different rounds [1]. This study aims to investigate whether this tournament scheduling strategy differs between top-ranked and lower-ranked players, as indicated by the number of tournaments they play. Specifically, players who performed well in higher-graded tournaments tend to take longer breaks between tournaments to recover fully, whereas those who performed less well in these tournaments tend to participate in more tournaments. It is proposed that only the first nine highest-scoring tournaments a player engages in within the past 52 weeks should be considered “countable tournaments”, highlighting a deliberate scheduling tactic that underscores the crucial role of adequate rest and strategic recovery periods in the ranking system for elite badminton competitors [22,23]. These events carry the most weight since they are the most recent and relevant in assessing a player’s current form and skill level [24]. Our findings suggest that lower-level athletes who employ structured recovery strategies, such as planned rest intervals and block periodization, can mitigate fatigue despite higher competition frequency.

This study examines if the top nine tournaments a player performed in over the previous 52 weeks can predict their ranking. The findings of our study support the hypothesis that top badminton players employ a strategic approach to their tournament scheduling to maximize their performance [22,23]. Moreover, this study also identifies the cutoff points that can distinguish top badminton players from their low-ranked counterparts while providing the quantitative matrices that underpin these criteria. Consistent with the findings of Cabello Manrique and González-Badillo (2003), who emphasize the competitive nature of badminton and the importance of tournament selection, this study investigates whether tournament scheduling strategy differs between top-ranked and lower-ranked players. Notably, players performing well in higher-graded tournaments often take longer breaks between competitions, aligning with Rahmat et al. (2022) who highlight the psychological impacts of intense competition, including the need for recovery to prevent burnout [25,26]. The data shows that players who participated in fewer tournaments but competed in better-graded competitions tend to perform better and earn more points, resulting in higher rankings. This finding is consistent with previous research highlighting the importance of tournament selection in optimizing performance and achieving higher rankings in badminton.

This study finds that top players tend to space out their tournaments to allow for adequate recovery time and to avoid excessive fatigue, burnout, or injury. Athletes who performed well in higher-graded games tend to take longer breaks between tournaments to perform well and recover fully. Furthermore, players who performed well in higher-graded tournaments tend to take longer breaks between tournaments to recover fully. By contrast, those who performed less well in these tournaments tend to participate in more tournaments to accumulate more points. Specifically, the findings discuss a study on top badminton player to determine the ideal time to space out their tournaments to avoid burnout and injury. This study sampled athletes ranked 1 to 50 and calculated the average and standard deviation of the interval weeks between tournaments. Results showed that the quenched average for top players to space out their tournaments was 5.04 weeks, while the annealed average was 5.06 weeks, providing adequate recovery time. Specifically, the average rest interval for top men’s singles players was 5.04 weeks (SD = 2.31), with a significant negative correlation between player rankings and rest interval durations (r = −0.31, *p* = 0.02). This finding suggests that higher-ranked players tend to take longer recovery periods compared to lower-ranked players. These results are illustrated in Figure 2, which presents the regression analysis outcomes. The findings presented in Figure 2 support the hypothesis that longer recovery periods are associated with better performance outcomes among top players.

By contrast, the top-ranked players had a higher standard deviation of the time interval between tournaments, indicating varied tournament schedules compared to lower-ranked players. However, this study could have found a better recovery time but highlighted that observing athletes’ real-life strategies can provide valuable insights into their recovery needs. By carefully selecting the intervals between tournaments, players can optimize their performance by ensuring adequate recovery time and minimizing the risk of injury or burnout [27]. The observed differences in rest intervals and tournament participation between higher- and lower-ranked players may partially reflect the influence of advanced planning models. Athletes with adequate time to adopt structured block or ATR-based preparation may be better equipped to prioritize recovery and focus on high-stakes tournaments, explaining their ability to sustain high performance.

Simulations of the ideal relationship between points earned and expected ranking in nine tournaments showed that increasing the number of tournaments tended to lead to more ideal results, as the simulation of ideal points earned corresponding to ideal ranking fits better with the actual data for men’s singles players participating in more than nine tournaments. A discrepancy between the actual and ideal data for the top 20 players indicates that the ideal relationship between points earned and expected ranking may vary for different performance levels. Specifically, the model perfectly fit the career progression of top-ranked men’s singles players, while it was less accurate for lower-ranked men’s singles players, revealing two distinct data clusters with a boundary around the 20th rank. Furthermore, the findings suggested that there is an ideal trade-off between the number of tournaments played and the points earned/ranking variances explained for increasing efficiency. The presented figure showing the prediction/simulation residuals at different numbers of tournaments played for both men’s and women’s players further highlights the importance of finding the ideal balance between the number of tournaments played and the points earned/ranking variances explained for maximizing efficiency [18]. These findings have important implications for sports organizations and athletes seeking to optimize their performance and ranking in tournaments [28]. Further research may help us to understand this trade-off better and improve the efficiency of ranking systems in sports.

In line with a previous finding, we compared the average number of tournaments played by the top 32 players in Olympic and non-Olympic years. This study selected high-ranked players using the criteria of 32 due to the absence of qualifying rounds in Super 1000 tournaments. There are only three annual tournaments with Super 1000 status: the All-England Open, the Indonesia Open, and the China Open. Access to these tournaments is exclusive to the most elite players, as they only have 32 players in each singles and doubles draw. However, this criterion was unsuitable for our model as it accurately represented the career progression of top-ranked players, but it needed to be more reliable for lower-ranked players. Two distinct data clusters were found for men’s singles players with a boundary around the 20th rank. This was also observed for women’s singles players, revealing two distinct data clusters with a boundary around the 12th rank. This study provides insight into the impact of the Olympic strategy that top-ranked men’s singles players employ in accumulating points during tournaments leading up to Olympic qualification and the overall points earned by lower-ranked men’s singles players. The findings indicate that the number of tournaments and the average number of tournaments played by top-ranked men’s singles players are higher during Olympic years compared to non-Olympic years. Additionally, the top 32 men’s singles players earned higher points during Olympic years than non-Olympic years, except for 2015; athletes ranking around 20 had fewer points in Olympic years than in non-Olympic years. Specifically, we observed that the top 20 players may be less affected by the strategy employed during the Olympic qualification year. The cutoff rankings among the top-ranked 20 men’s singles players within the ranking can be considered top-ranked, while the rest of the lower-ranked players can be considered top players. This observation was also found in women’s singles players, suggesting two distinct data clusters with a boundary around the 12th rank. This observation highlights that the Olympic strategy employed by top-ranked players can significantly affect the overall points earned by lower-ranked players and their rankings in the tournament bracket. Based on the results presented in Figure 2, which demonstrate a significant inverse correlation between ranking and rest intervals (r = −0.31, *p* = 0.02), we infer that top-ranked players strategically prioritize longer recovery periods to maintain peak performance. This aligns with previous studies that emphasize the importance of adequate recovery in optimizing athlete performance (Cabello Manrique and González-Badillo, 2003). By contrast, lower-ranked players may prioritize frequent tournament participation to accumulate ranking points, potentially at the cost of recovery. The figures presented in this study effectively support and explain the findings. The results of this study have important implications for athletes, coaches, and organizations preparing and participating in tournaments leading to Olympic qualification. The findings suggest the need for a more equitable distribution of tournaments and a re-evaluation of the Olympic strategy employed by top-ranked players to ensure a level playing field for all athletes.

While providing valuable insights into the tournament scheduling strategies of top-level badminton players, this study also has certain limitations that should be acknowledged. Primarily, the analysis was confined to the top 50 men’s and women’s singles players. This choice was driven by two main factors: firstly, data accessibility constraints limited our ability to comprehensively gather information on all players, leading us to focus on the top 50 where data was more robust and reliable. Secondly, the distribution of tournament points, especially in high-grade events, like the Olympics and the World Championships, or Grade 2 tournaments, such as the Super Series, varies significantly for players ranked below 50. Concentrating on the top 50 ensured that our dataset was concentrated and relevant to the objectives of this study. However, this focus limits our findings’ generalizability to the entire spectrum of top badminton players. Future research could expand on our work by exploring tournament scheduling strategies across a broader range of player rankings, including those outside the top 50, and possibly extend the investigation to other sports to compare and contrast strategic scheduling patterns. Moreover, another limitation of the current study is the lack of control for player injuries, which may affect tournament participation. As injuries can cause players to withdraw from tournaments or limit their ability to compete at their best, future studies should incorporate this factor where possible to provide a more accurate representation of the influence of tournament participation on performance. These explorations would offer a more comprehensive understanding of the impact of tournament scheduling on player performance and ranking across different levels of sport.

## 5. Conclusions

In conclusion, this study investigated whether top-level badminton players strategically schedule tournament participation to optimize their performance and ranking. The findings support the hypothesis that players who compete in fewer tournaments but in better-graded competitions tend to perform better and earn more points, resulting in higher rankings. Top players tend to space out their tournaments to avoid burnout, excessive fatigue, or injury. Players who perform well in higher-graded tournaments take longer breaks between tournaments to recover fully. Additionally, this study suggests an ideal trade-off between the number of tournaments played and the points earned/ranking variances explained for increasing efficiency. However, the ideal relationship between points earned and expected ranking may vary for different performance levels. This study also highlights the impact of the Olympic strategy employed by top-ranked players on the overall points earned by lower-ranked players and the need for a more equitable distribution of tournaments. While this study focused on analyzing tournament schedules and performance outcomes, the principles of block periodization and ATR training models offer valuable context in understanding how athletes might optimize preparation and recovery for competitions. Future research should investigate the role of these structured preparation strategies in tournament selection, recovery management, and overall performance. Understanding how these approaches influence athletes’ ability to balance competition frequency and recovery could provide deeper insights into the factors that differentiate top-ranked players from their lower-ranked counterparts. For athletes, the results indicate the importance of tournament scheduling to maximize performance while minimizing risks of injury and burnout [4,5,6,7,8]. Federations and coaches can leverage these insights to design tailored training and recovery plans, ensuring athletes maintain peak physical and mental readiness for high-stakes competitions. For example, implementing periodic training programs aligned with tournament schedules can help athletes sustain performance across a demanding competitive season. Finally, this study offers a more in-depth understanding of the criteria for distinguishing elite players from their low-ranked counterparts. These findings have significant implications for sports organizations, athletes, and coaches seeking to optimize their performance and ranking in tournaments leading to Olympic qualification. This study concludes that the top 20 are for men’s singles players, and the top 12 are for women’s singles players. Future research can help better understand the trade-off and improve the efficiency of ranking systems in sports.

## Data Availability

The original contributions presented in this study are included in the article. Further inquiries can be directed to the corresponding author.
